# Waist circumference cut-off points to identify major cardiovascular events and incident diabetes in Latin America: findings from the prospective Urban rural epidemiology study Colombia

**DOI:** 10.3389/fcvm.2023.1204885

**Published:** 2023-10-30

**Authors:** Jose P. Lopez-Lopez, Ana María Gonzalez, Paola Lanza, Daniel Martinez-Bello, Diego Gomez-Arbelaez, Johanna Otero, Daniel D. Cohen, Maritza Perez-Mayorga, Angel A. Garcia-Peña, Sumathy Rangarajan, Salim Yusuf, Patricio Lopez-Jaramillo

**Affiliations:** ^1^ MASIRA Research Institute, Universidad de Santander (UDES), Bucaramanga, Colombia; ^2^ Cardiology Unit, Department of Internal Medicine, Hospital Universitario San Ignacio, Pontificia Universidad Javeriana, Bogotá, Colombia; ^3^ Medicine School, Universidad Militar Nueva Granada, Clínica Marly, Bogotá, Colombia; ^4^ Department of Health Research Methods, Evidence, and Impact, McMaster University and Population Health Research Institute, Hamilton Health Sciences and McMaster University, Hamilton, ON, Canada

**Keywords:** abdominal obesity, waist circumference, major cardiovascular events, diabetes, Latin America

## Abstract

**Background:**

Abdominal obesity (AO) indirectly represents visceral adiposity and can be assessed by waist circumference (WC) measurement. In Latin America, cut-off points for the diagnosis of AO are based on Asian population data. We aim to establish the WC cut-off points to predict major cardiovascular events (MACE) and incident diabetes.

**Methods:**

We analyzed data from the cohort PURE study in Colombia. WC cut-off points were defined according to the maximum Youden index. Multivariate logistic regression was used to obtain associations between WC and MACE, diabetes, and cumulative incidence of outcomes visualized using Kaplan-Meier curves.

**Results:**

After a mean follow-up of 12 years, 6,580 individuals with a mean age of 50.7 ± 9.7 years were included; 64.2% were women, and 53.5% were from rural areas. The mean WC was 85.2 ± 11.6 cm and 88.3 ± 11.1 cm in women and men, respectively. There were 635 cases of the MACE composite plus incident diabetes (5.25 events per 1,000 person-years). Using a cut-off value of 88.85 cm in men (sensitivity = 0.565) and 85.65 cm in women (sensitivity = 0.558) resulted in the highest value for the prediction of the main outcome. These values were associated with a 1.76 and 1.41-fold increased risk of presenting the composite outcome in men and women, respectively.

**Conclusions:**

We defined WC cut-off points of 89 cm in men and 86 cm in women to identify the elevated risk of MACE and incident diabetes. Therefore, we suggest using these values in cardiovascular risk assessment in Latin America.

## 1. Introduction

Cardiovascular disease (CVD) remains the principal cause of morbidity and mortality in Latin America and worldwide (1, 2). It has been demonstrated that modifiable risk factors explain approximately 70% of major cardiovascular events (MACE) (2–4). Among these risk factors, abdominal obesity (AO) contributes to 15% of the population attributable fraction, a higher percentage than other risk factors such as smoking or dyslipidemia (1). AO indirectly represents visceral adipose tissue accumulation and can be quantified by various methods, most commonly the simple and easily applicable waist circumference (WC) measurement using a tape measure. WC measuring is usually taken at the midpoint between the inferior costal margin and the iliac crest. Large-scale observational data shows that as WC increases, the number of cardiovascular risk factors, cardiovascular mortality, and all-cause mortality increases (5–8). In addition, from a metabolic perspective, crosstalk of inflammatory signals between visceral adiposity and insulin-dependent cells leads to an increased risk of developing diabetes (9). Several studies in different geographical populations and ethnicities have confirmed the association between AO and diabetes (10, 11).

A high WC is also a cornerstone component of the metabolic syndrome diagnosis. The International Diabetes Federation (IDF) determined that the WC cut-off points used for AO should be specific to geographic location (12), with distinct values for Europe, the United States, and Southeast Asia. With respect to Latin America, including Central and South America, IDF recommends that until more information is available, cut-off points for Asian countries (≥80 cm in women and ≥90 cm in men) be used. This recommendation was made more than 15 years ago and is still applied in the clinical setting, but it has also led to multiple studies in Latin America aimed at clarifying associations in this population. Most of these studies were cross-sectional and showed that specific WC cut-off points are associated with the intra-abdominal fat volume measured by computed tomography or with metabolic and inflammatory alterations (13–18). However, these measured outcomes have generated debate about their applicability in clinical prognosis. In Latin America, there also remains a paucity of follow-up cohort/prospective data upon which to define region-specific WC cut-off points that predict MACE and diabetes. Therefore, this study aims to establish the WC cut-off points with the greatest capacity to predict MACE and incident diabetes in the Colombian population of the Prospective Urban Rural Epidemiology (PURE) study.

## 2. Materials and methods

### 2.1. Study design, population, sociodemographic and clinical data

The PURE study is a large-scale prospective multinational cohort study including high, middle, and low-income countries. The study design, including sample size, has previously been reported (19). For the present analysis, we included 6,580 with complete data. A multistage convenience sample survey was used, and a representative sample of households was recruited using a community-sampling framework. Households were eligible if at least one member was 35–70 years old and if the members intended to continue living at that address for 4 years or more. The participants were selected from urban and rural communities in 11 departments of the country. After informed consent was obtained, questionnaires were applied to identify sociodemographic, clinical, and anthropometric data. Demographic characteristics such as area of residence and socioeconomic characteristics such as educational level was recorded. In addition, the history of cardiometabolic events and associated risk factors were examined. Current smokers were defined as those who reported having used a tobacco product in the past 12 months. Current alcohol consumption was defined as those who declared alcohol consumption in the last year. Every year, the participants are followed up by telephone to inquire about their vital status and the outcomes. Every three years, in addition to the telephone contact, a broader questionnaire is made inquiring about lifestyles and access to health services. In addition, anthropometric and laboratory measurements are made according to the protocol. For the analysis, we used the baseline measurements (between 2005 and 2009) and presential follow-up visits (mean follow-up of 12 ± 2.3 years) to identify cardiometabolic outcomes.

### 2.2. Anthropometric measures

Anthropometric measurements were acquired following the PURE standardized protocol. Body weight was obtained using a digital scale, ensuring that the patient wore light clothing, and height was obtained using a tape measure, approximating each measurement to the nearest centimeter. The patient was instructed to be barefoot for both measurements. WC and hip circumference were measured with tape on the patient's skin. WC was considered the smallest circumference between the lower costal margin and the upper margin of the iliac crest. The hip circumference was measured at the level of the greater trochanters. Body mass index (BMI) by dividing body weight (in kilograms) by height (in meters) squared.

### 2.3. Outcomes

The main MACE composite outcome was defined as the occurrence of cardiovascular death, myocardial infarction, stroke, or heart failure, whichever came first. Myocardial infarction was defined as typical or symptoms suggestive of a heart attack according to the medical professional associated with electrocardiogram changes or changes in biomarkers; stroke as an acute focal neurological deficit diagnosed by a physician and thought to be of vascular origin (without another case such as a brain tumor) with signs and symptoms lasting ≥24 h; finally, heart failure was defined as signs (rales, increased jugular venous pressure, or ankle edema) or symptoms (paroxysmal nocturnal dyspnea, dyspnea at rest, or ankle edema) of congestive heart failure and one or both of the following: radiological studies of pulmonary congestion, treatment of heart failure with diuretics. Diabetes was defined as fasting blood glucose >126 mg/dl or a history of diabetes (self-reported) or current diabetes treatment. The outcome variables were taken with a cut-off date of February 28, 2021.

### 2.4. Statistical analysis

Continuous variables were presented as means and standard deviations. Categorical variables were presented as absolute frequencies and percentages. The analysis of variance (ANOVA) method was used to examine differences between continuous variables and Pearson's chi-square test between categorical variables. First, WC cut-off points for maximum sensitivity and specificity for the presence or absence of the time to event outcomes (MACE, fatal and non-fatal stroke, fatal and non-fatal myocardial infarction, fatal MACE, diabetes and the MACE and diabetes composite) were determined with the maximum Youden index. The ROC curves and the integrated area under the curve iAUC (20) were estimated for censored survival data at 12 years of follow-up using the risksetROC library version 1.0.4.1. Second, Kaplan-Meier curves were used to visualize the cumulative incidence of the composite outcome splitting by the previously established cut-off points. Third, the association between the WC cut-off points and the composite outcome was analyzed using the Cox proportional hazards model with random effects given by the Colombian provinces estimating hazard ratio (HR) and 95% confidence intervals (CI), using the coxme version 2.2–18.1. All statistical analyses were conducted using the R software version 4.2.2. All *p* values were two-sided, and the significance level was 5%.

## 3. Results

At the baseline, the overall mean age was 50.7 ± 9.7 years, 4,845 (64.2%) participants were women, and 53.5% of the population were from rural areas. In addition, women had a WC mean of 85.2 ± 11.6 cm, while men had 88.3 ± 11.1 cm. Other baseline characteristics are shown in Table 1. During a median follow-up of 12 ± 2.3 years, there were 419 events of the MACE composite, 138 cardiovascular deaths, 184 incident cases of myocardial infarction, 88 incident cases of stroke, 304 cases of incident diabetes, and 635 cases of the MACE composite plus incident diabetes (5.25 events per 1,000 person-years). The assessment for the optimal performance of WC cut-off points according to sex to identify individuals at risk of composite MACE and incident diabetes is shown in Table 2.

**Table 1:** Baseline characteristics of study participants.

Characteristics	Total	Female	Male	*p* value
Participants, *n* (%)	7,552	4,845 (64.2)	2,707 (35.8)	
Ages, years, mean (SD)	50.78 (9.74)	50.57 (9.60)	51.15 (9.96)	0.018
Location				
Rural, *n* (%)	4,043 (53.5)	2,378 (49.1)	1,665 (61.5)	<0.001
Urban, *n* (%)	3,509 (46.5)	2,467 (50.9)	1,042 (38.5)	
Education level				
Low, *n* (%)	4,988 (66.2)	3,142 (65.0)	1,846 (68.2)	0.003
Middle, *n* (%)	1,480 (19.6)	1,004 (20.8)	476 (17.6)	
High, *n* (%)	1,072 (14.2)	688 (14.2)	384 (14.2)	
Cigarette smoking				
Ex -smoker, *n* (%)	1,561 (20.7)	705 (14.6)	856 (31.7)	<0.001
Current, *n* (%)	1,045 (13.9)	446 (9.2)	599 (22.2)	
Never, *n* (%)	4,931 (65.4)	3,685 (76.2)	1,246 (46.1)	
Consumption of alcohol				
Ex -drinker, *n* (%)	1,184 (15.7)	568 (11.7)	616 (22.8)	<0.001
Current, *n* (%)	2,128 (28.2)	784 (16.2)	1,344 (49.6)	
Never, *n* (%)	4,235 (56.1)	3,488 (72.1)	747 (27.6)	
Hypertension, *n* (%)	2,846 (37.7)	1,869 (38.6)	977 (36.1)	0.037
Diabetes, *n* (%)	424 (5.6)	286 (5.9)	138 (5.1)	0.162
Ischemic cardiopathy, *n* (%)	183 (2.4)	118 (2.4)	65 (2.4)	0.985
Heart failure *n* (%)	115 (1.5)	78 (1.6)	37 (1.4)	0.466
Stroke *n* (%)	113 (1.5)	82 (1.7)	31 (1.1)	0.076
Other cardiovascular diseases, *n* (%)	105 (1.4)	77 (1.6)	28 (1.0)	0.061
Waist circumference, mean (SD)	86.3 (11.5)	85.2 (11.6)	88.3 (11.1)	<0.001
Hip circumference, mean (SD)	97.6 (10.1)	99.3 (10.2)	94.7 (9.2)	<0.001
Body mass index, mean (SD)	26.2 (4.6)	26.8 (4.9)	25.2 (4.1)	<0.001

SD, standard derivation; *p* value: analysis of variance (ANOVA) of one factor (continuous variables), and Pearson's chi-square test (categorical variables).

**Table 2:** Optimal cut-off points for waist circumference to identify Major cardiovascular events and incident diabetes. *n* = 6,580.

	Proposed cutt-off point (cm)	AUC	Sensitivity	Specificity	Youden index	Sensitivity with IDF cut-off point^b^	Specificity with IDF cut-off point^b^	Youden index
Male								
MACE^a^	88.2	0.571	0.526	0.574	0.100	0.455	0.643	0.098
Incident diabetes	90.9	0.699	0.619	0.666	0.285	0.650	0.634	0.284
MACE + diabetes	88.85	0.613	0.565	0.595	0.159	0.517	0.641	0.158
Female								
MACE^a^	85.55	0.596	0.545	0.591	0.136	0.729	0.391	0.121
Incident diabetes	86.65	0.646	0.578	0.629	0.207	0.777	0.393	0.176
MACE + diabetes	85.65	0.613	0.558	0.602	0.160	0.737	0.404	0.141

MACE, Mayor adverse cardiovascular events; AUC, area under the curve; Youden index, sensitivity + 1—specificity.

^a^ MACE: composite of cardiovascular death, myocardial infarction, heart failure, and stroke.

^b^ IDF cut-off point: 90 cm in men and 80 cm in women.

**Figure 1:**
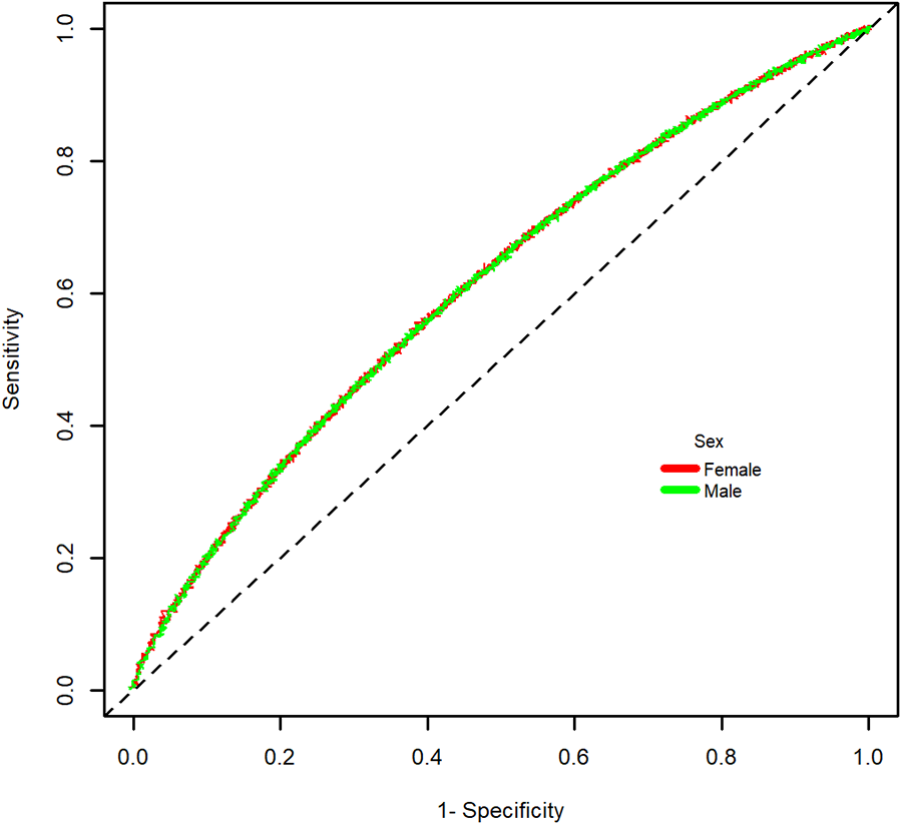
ROC curve for major cardiovascular events or incident diabetes from waist circumference.

**Figure 2:**
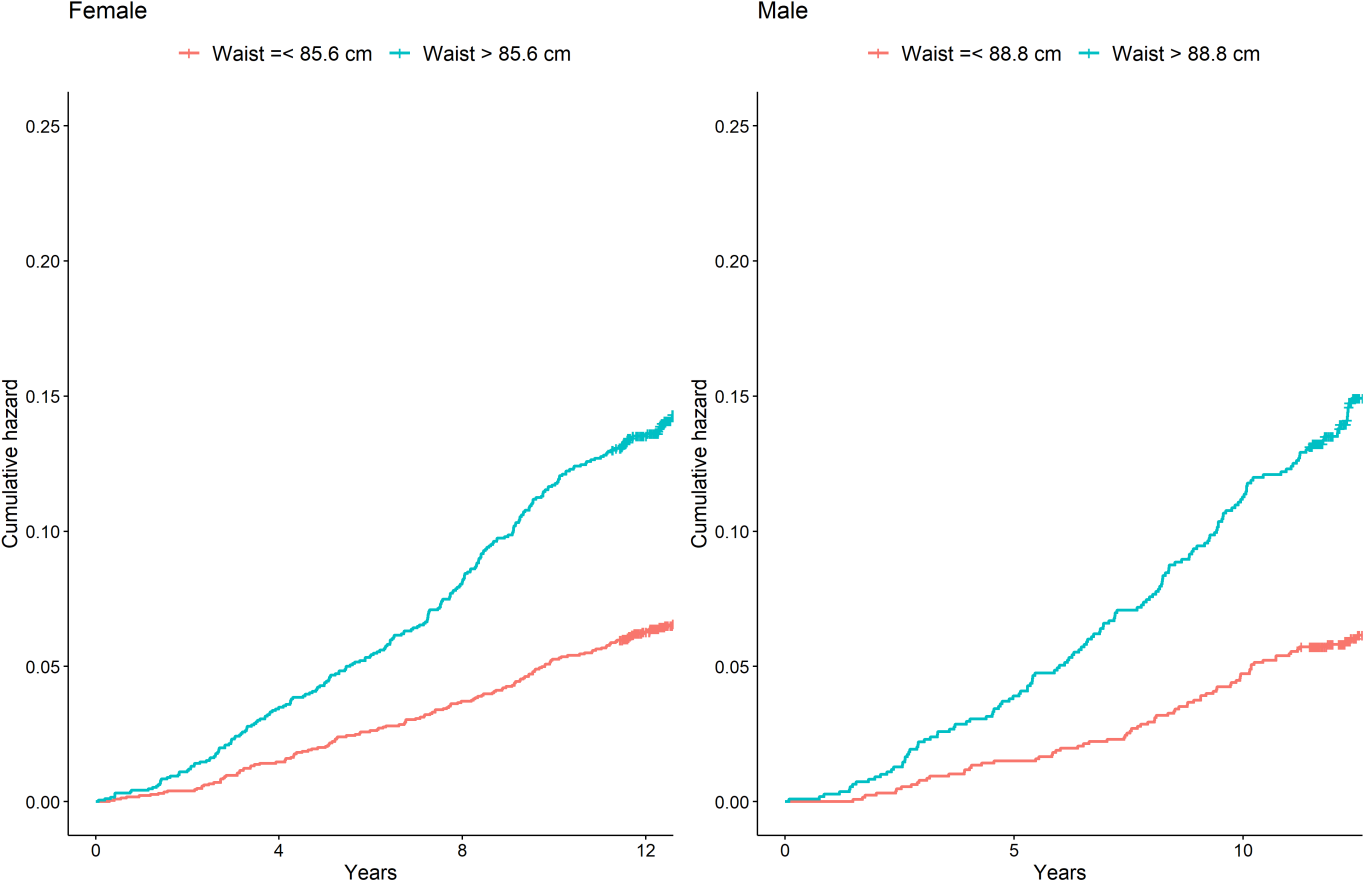
Cumulative incidence of major cardiovascular events and incident diabetes by waist circumference higher or lower that 89 cm in men and 86 cm in women. Shown are Kaplan–Meier event curves for the main composite outcome of cardiovascular death, myocardial infarction, ischemic stroke, and incident diabetes.

With a cut-off value of 88.85 cm in men (sensitivity = 0.565), and 85.65 cm in women (sensitivity = 0.558) the Youden index was the highest for the identification of the composite of MACE plus incident diabetes (1,233 in men and 1,203 in women). The ROC curve for the main outcome is shown in Figure 1. The AUC was 0.613 in men and 0.613 in women. In addition, the optimal WC cut-off points were positively associated with the risk of presenting the outcome composite (Table 3). After establishing a WC cut-off value of 89 cm, men above this value had a 1.76-fold increased risk of presenting the composite outcome (HR = 1.76, 95% CI: 1.26–2.45). A WC cut-off value greater or equal to 86 cm in women had a 1.41-fold higher risk (HR = 1.46, 95% CI: 1.09–1.83) (Figure 2).

**Table 3:** Association between optimal waist circumference cutoff point with Major cardiovascular events and diabetes.

	Male				Female			
	Univariate		Multivariate^a^		Univariate		Multivariate^a^	
	HR (CI95%)	*p* value	HR (CI95%)	*p* value	HR (CI95%)	*p* value	HR (CI95%)	*p* value
MACE^b^	1.77 (1.29–2.42)	<0.001	1.55 (1.04–2.30)	0.031	1.91 (1.47–2.47)	<0.001	1.24 (0.89–1.72)	0.200
Cardiovascular mortality	1.35 (0.83–2.17)	0.220	1.10 (0.59–2.05)	0.760	2.82 (1.71–4.67)	<0.001	1.73 (0.94–3.21)	0.080
Myocardial infarction	1.73 (1.11–2.69)	0.015	1.54 (0.88–2.70)	0.130	2.60 (1.70–3.97)	<0.001	1.60 (0.95–2.71)	0.080
Stroke	1.77 (0.92–3.44)	0.089	1.33 (0.57–3.11)	0.500	1.61 (0.92–2.81)	0.092	1.26 (0.62–2.55)	0.530
Incident diabetes	5.54 (3.39–9.06)	<0.001	3.58 (2.07–6.18)	<0.001	2.76 (2.07–3.66)	<0.001	1.56 (1.10–2.22	0.013
MACE + Diabetes	2.36 (1.79–3.11)	<0.001	1.76 (1.26–2.45)	0.001	2.32 (1.88–2.86)	<0.001	1.41 (1.09–1.83)	0.009

MACE, Mayor adverse cardiovascular events; IC95%, confidence interval 95%; HR, hazard ratio; *p*-value, Univariate and multivariate regression models of proportional hazards with random effects.

^a^ Models were adjusted for age, location (urban/rural), level of education, tobacco and alcohol consumption, history of hypertension and body mass index.

^b^ MACE, composite of cardiovascular death, myocardial infarction, heart failure, and stroke.

## 4. Discussion

### 4.1. Key findings

In this analysis of a prospective cohort study with more than 6,500 Colombian adults and a mean follow-up of 12 years, we demonstrated that the optimal WC cut-off point to identify the risk of the composite of MACE and incident diabetes is 89 cm in men and 86 cm in women. Since 2006, when the IDF metabolic syndrome guidelines recommended taking WC cut-off points for determining AO derived from the population of Southeast Asia in the absence of data in Latin America, multiple studies have been carried out to identify the optimal value for our population.

The Peruvian Study of Cardiovascular Disease (PREVENCION) (13) in 1,439 adults showed that cut-off points of 97 cm in men and 87 cm in women were associated with subclinical atherosclerotic disease (defined by the thickness of carotid intima-media) or overt CVD including diabetes as an equivalent of vascular disease risk. The values in the present study resemble those found primarily in women; however, PREVENCION was a cross-sectional study in an Andean population of southern Peru at 2,335 meters above sea level, making it difficult to generalize the results. In contrast, the PURE Colombia study was carried out in 11 departments with different altitudes and representativeness of roughly 51% of the Colombian population from different ethnic groups. The Brazilian Longitudinal Study of Adult Health (ELSA-Brasil) also evaluated the cut-off points of WC in other Latin American populations. Their results align with ours, especially in women, where the optimal cut-off points were the same (86 cm). Unlike our study, in the ELSA-Brasil study, the outcomes assessed were intermediate (metabolic parameters), contrary to MACE and diabetes evaluated in the present study. In addition, the study mentioned above had a cross-sectional nature. In contrast, the data from PURE-Colombia have a prospective average follow-up of 12 years, enabling the evaluation of “hard” outcomes (21).

Recently, the cross-sectional Hispanic Community Health Study/Study of Latinos (HCHS/SOL) evaluated 16,415 self-identified Hispanic/Latino subjects residing in the United States (14). The results suggested an optimal cut-off point of 102 cm and 97 cm in men and women, respectively, to discriminate the risk of ischemic heart disease. Men's cut-off points are like those recommended by the IDF for the non-Hispanic population of the United States. We can therefore infer an environmental influence on the risk of metabolic and CVD; and in our socioeconomic conditions, distinct from those of high-income countries, using a higher cut-off point could lead to underdiagnosis or late identification of this cardiovascular risk factor (22).

In Colombia, in males national air force staff, a WC cut-off point of 88 cm was associated with cardiometabolic alterations, including dyslipidemia and elevation of the acute phase reactant C-reactive protein (15). Additionally, the Diagnóstico del Riesgo Cardiovascular Global study in Medellín determined practically identical values as that of our study (92 cm in men and 84 cm in women) to identify insulin resistance through the HOMA insulin resistance index (IR -HOMA) (16). The main limitation of these studies is their cross-sectional nature and intermediate outcomes. Cut-off points of 94 cm in men and 90 cm in women have been suggested based in the area of visceral adipose tissue measured with computerized tomography scans (17). Although that area is associated with metabolic alterations that can predict cardiometabolic disease, data assessing MACE outcomes is scarce, highlighting the importance of our results. The results presented are also comparable to the recommendations made by the IDF, particularly in men. Potentially, this could be related to the sociodemographic features that influence the appearance of CVD in our population. Recent data from an analysis of the PURE study in Southeast Asia and South America showed that socioeconomic and psychosocial risk factors, such as low education, contribute a significant population-attributable risk fraction for CVD and general mortality (1, 23). In addition, the early detection of cardiovascular risk factors, in this case, a lower cut-off point in men, is of substantial importance, considering that for overall mortality, the age-standardized male-female ratio in South America is 1,72. In women, a cut-off point of 86 cm, higher than that recommended by the IDF, seems to be an appropriate value, taking into account that large observational studies such as the International Day for Evaluation of Abdominal adiposity (IDEA) showed that a cut-off point of 80 cm overestimates the definition of AO (24). The last Latin American consensus on the management of hypertension in patients with diabetes and metabolic syndrome (25) recommended carrying out a cohort study to determine the value of the abdominal perimeter best associated with hard outcomes, such as diabetes and CVD. The results of the present study that follows up this recommendation demonstrate that a WC cut-off points of 89 cm in men and 86 cm in women are the best in our population to identify the risk of diabetes and CVD.

### 4.2. Study limitations

However, our study has potential limitations. While the data may be representative of the Colombian population, these results may not be generalizable to the rest of Latin America. In addition, 65% of the population included were women, which limits the sample's representativeness. However, as mentioned, there is a paucity of prospective cohort studies evaluating the relationship between AO and CVD in this population. In addition, the WC measurement cannot differentiate between visceral and subcutaneous adiposity, especially in people with higher BMIs, so different imaging techniques to quantify visceral adiposity and predict cardiovascular risk should be useful. However, the measurement of WC with tape is an inexpensive, widely available tool that is applicable in clinical assessment. Although cardiovascular epidemiological studies such as INTERHEART (3) and INTERSTROKE (4) demonstrated that in the Latin American population, AO objectively measured by the waist-hip ratio is significantly associated with the appearance of the first event of myocardial infarction or stroke, the present analysis shows that a single measurement of WC has a similar association with MACE; therefore, from a practical point of view, a single measurement may be recommended.

## 5. Conclusions

In conclusion, this study reinforces the association between AO determined by WC and the risk of CVD and diabetes for men (HR = 1.76, 95% CI: 1.26–2.45) and women (HR = 1.46, 95% CI: 1.09–1.83). Furthermore, we determined WC cut-off points of 89 cm in men and 86 cm in women as the best to identify this risk. Therefore, we suggest that these values should be used in patients' comprehensive cardiovascular risk assessment in Latin America.

## Data Availability

Individual-level data will not be shared because PURE is an ongoing cohort study. Requests for aggregate data will be considered on a case-by-case basis on receipt of a reasonable request.
